# Intense/obsessional interests in children with gender dysphoria: a cross-validation study using the Teacher’s Report Form

**DOI:** 10.1186/s13034-017-0189-9

**Published:** 2017-09-25

**Authors:** Kenneth J. Zucker, A. Natisha Nabbijohn, Alanna Santarossa, Hayley Wood, Susan J. Bradley, Joanna Matthews, Doug P. VanderLaan

**Affiliations:** 1 Department of Psychiatry, University of Toronto, Toronto, ON M5T 1R8 Canada; 2 Department of Psychology, University of Toronto Mississauga, Mississauga, ON Canada; 3 Psychological Services, Toronto District School Board, Toronto, ON Canada; 4 Underserved Populations Research Program, Child, Youth and Family Division, Centre for Addiction and Mental Health, Toronto, ON Canada

**Keywords:** Gender dysphoria, Autism Spectrum Disorder, Teacher’s Report Form, Equifinality, DSM-5

## Abstract

**Objective:**

This study assessed whether children clinically referred for gender dysphoria (GD) show symptoms that overlap with Autism Spectrum Disorder (ASD). Circumscribed preoccupations/intense interests and repetitive behaviors were considered as overlapping symptoms expressed in both GD and ASD.

**Methods:**

To assess these constructs, we examined Items 9 and 66 on the Teacher’s Report Form (TRF), which measure obsessions and compulsions, respectively.

**Results:**

For Item 9, gender-referred children (n = 386) were significantly elevated compared to the referred (n = 965) and non-referred children (n = 965) from the TRF standardization sample. For Item 66, gender-referred children were elevated in comparison to the non-referred children, but not the referred children.

**Conclusions:**

These findings provided cross-validation of a previous study in which the same patterns were found using the Child Behavior Checklist (Vanderlaan et al. in J Sex Res 52:213–19, 2015). We discuss possible developmental pathways between GD and ASD, including a consideration of the principle of equifinality.

## Background

Children with a DSM-5 diagnosis of gender dysphoria (GD) [Gender Identity Disorder of Childhood in DSM-III and III-R and Gender Identity Disorder (GID) in DSM-IV] have a marked incongruence between the gender they have been assigned to at birth and their experienced/expressed gender [1].^1^ The DSM-5 indicators for the diagnosis, as in DSM-III and DSM-IV, include an array of sex-typed behaviors (e.g., toy and activity interests, dress-up play, roles in fantasy play, etc.) that often signal a strong identification with the other gender. Over three decades ago, Coates [2] reported the clinical impression that at least some boys with GD appeared to show an intense, if not obsessional, interest in gender-related themes, as manifested in their surface behaviors and in fantasy play, and in their responses during projective testing such as the Rorschach [3] (for a recent clinical example, see Saketopoulou [4]. It is unclear, however, whether these patterns of behavior are simply an “inverted” instance of the intense gender-related interests and behaviors seen in typically-developing children [5, 6] or represent something that is qualitatively distinct or, at least, at the extreme end of a quantitative spectrum.

One relatively recent line of research, stimulated by a series of clinical case reports and one internet-recruited sample (of children, adolescents, and adults), has pointed to a possible link between GD and Autism Spectrum Disorder (ASD) or at least traits of ASD [7–19]. Using a structured diagnostic interview schedule, dimensional measures, or chart review, several studies have reported, compared to normative samples, an overrepresentation of either ASD or ASD traits among clinic-referred children and/or adolescents [20–23] or adults [24, 25] with a diagnosis of GID/GD (for an internet-recruited sample, see also Kristensen and Broome [26] (for reviews, see Glidden et al. [27], Strang et al. [28], van der Miesen et al. [29], and van Schalkwyk et al. [30] ).

One potential explanation for the putative link between GD and ASD is the intense focus on, or an obsessional interest in, specific activities [31, 32]. Such interests relate to the DSM-5 ASD criterion pertaining to highly restricted and fixated interests. For example, it is conceivable that children with ASD who form intense and focused attention to cross-sex objects or activities may then begin to express other characteristics of GD (e.g., see Strang et al. [33]). Conversely, GD may give rise to such interests and obsessions, leading to a clinical presentation consistent with ASD. In order to appraise these two proposed pathways, however, the first step would be to determine empirically if, in fact, children with GD manifest an elevated pattern of intense interests and obsessions.

To our knowledge, only two studies have focused on a possible elevation in obsessional/repetitive interests and behaviors in GD children using dimensional metrics. Skagerberg et al. [23] used the Social Responsiveness Scale (SRS) in a mixed sample of 166 children and adolescents and found an elevation on the “Autistic Mannerisms” subscale completed by the parents [now labeled “Restricted Interests and Repetitive Behaviors” (RIRB) on the SRS-2] [34] compared to a normative sample. However, two methodological issues call for some caution in appraising the results. First, the participation rate was only 46%, which may represent a threat to the internal validity of the sample [35]. Second, a clinic-referred comparison group, consisting of children/adolescents referred for other clinical problems, was not included. Thus, it is not clear if the elevation on the Autistic Mannerisms subscale is specific to children/adolescents referred for gender dysphoria or characteristic of clinic-referred children/adolescents in general.

Taking advantage of a large “archival” data set, VanderLaan et al. [36] analyzed two items on the Child Behavior Checklist (CBCL) [37] pertaining to obsessionality and repetitive behavior: Item 9 (“Can’t get his/her mind off certain thoughts; obsessions”) and Item 66 (“Repeats certain acts over and over; compulsions”) in a sample of 534 children referred clinically for gender identity concerns, 419 siblings, and 1201 referred and 1201 non-referred children from the CBCL standardization sample [37], with an age range of 3–12 years.^2^ For both items, parental responses were dichotomized as either present (“Somewhat or sometimes true”/“Very true or often true”) or absent (“Not true”). In their study, the parental participation rate was over 90% for the gender-referred sample.

For Item 9, the percentage of mothers of the gender-referred children who endorsed it (62.4%) was significantly greater than that of their siblings (22.2%) and significantly greater than the ratings of the mothers of both the referred (48.7%) and non-referred (21.9%) children from the CBCL standardization sample (odds ratios, with a 95% CI ranged from 1.66 to 10.96). The percentage of mothers of the referred children who endorsed it was also significantly greater than the ratings for the siblings and of the non-referred children. For Item 66, the percentage of mothers of the gender-referred children who endorsed it (25.3%) was significantly greater than that of their siblings (8.2%) and the ratings of the non-referred children (5.4%) (odds ratios ranged from 3.04 to 6.77), but not of the referred children (24.9%), who also had higher endorsement ratings than the siblings of the gender-referred children and of the non-referred children. Thus, in this study, there was evidence for both specificity and non-specificity for these two behaviors: On the one hand, both the gender-referred children and the referred children were elevated on both items compared to the siblings and non-referred children (non-specificity); on the other hand, a greater percentage of the gender-referred children than the referred children were elevated on Item 9, evidence for at least partial specificity.

For the gender-referred children and their siblings, it was also possible to code qualitatively the reasons that the mothers endorsed these two items. A two-option coding scheme classified the reasons as either gender-related (e.g., “Cinderella” for Item 9) or non-gender-related (e.g., “killing”). For Item 9, VanderLaan et al. [36] found that gender-related themes were significantly more common for the gender-referred boys than that of the male siblings, but the difference between the gender-referred girls and that of the female siblings was not significant (possibly due to low power because of the smaller sample size). For Item 66, there was no significant difference in gender-related themes for the gender-referred children and their siblings.

The purpose of the present study was to cross-validate the VanderLaan et al. [36] findings for these two items using teacher ratings on the Teacher’s Report Form [38] to see if teachers would also report elevations in gender-referred children when compared to both referred and non-referred children in the TRF standardization sample [39].^3^

## Methods

### Participants

Between 1986 and 2013, TRFs were obtained for 386 children (304 boys; 82 girls) who were referred to, and then assessed in, a specialty gender identity service for children, housed within a child psychiatry program at an academic health science center. The children had a mean age of 7.77 years (SD = 2.41). All of the children met DSM-III, DSM-IV or DSM-5 criteria for GID/GD or were subthreshold for the diagnosis (e.g., Gender Identity Disorder NOS). During this time period, TRFs were not available for an additional 145 gender-referred children. The main reasons for this were: the parents did not want the teacher to complete the TRF (because of concerns about privacy/confidentiality); a TRF was mailed to the teacher/school, but it was not returned; the child was too young for the TRF to be administered (e.g., not yet in school); the child was being home-schooled; or, the family chose not to complete the assessment so the TRF was not sent to the teacher.^4^

For comparative purposes, we used the TRF referred (498 boys; 467 girls) and non-referred (498 boys; 467 girls) standardization samples for children ages 6–12 years from Achenbach and Rescorla [39]. As reported by Achenbach and Rescorla, the referred sample was obtained from various mental health and special educational settings, primarily in the U.S., heterogeneous with regard to DSM diagnoses. The non-referred sample was obtained from the 1999 National Survey of Children, Youths, and Adults conducted between February 1999 and January 2000. Parents who completed the CBCL were asked for permission to mail a TRF to one of their child’s teachers, who received $10 in compensation for participation. Children were included in the non-referred sample if they had not received professional help for behavioral, emotional, substance use, or developmental problems in the preceding 12 months [39, pp. 75–76]. The referred and non-referred samples were matched for gender, age, socioeconomic status, and ethnicity [39, pp. 75–76, p. 109].

### Measures

For both Items 9 and 66, teacher responses were dichotomized where 0 = 0 and 1 or 2 = 1. Using the parental data from our previous study for the gender-referred sample [36], we calculated mother–teacher and father–teacher correlations for both items using the continuous 0 to 2 coding system. For the gender-referred children, we recorded the comments provided by the teacher if the items were scored either as a 1 (“somewhat or sometimes true”) or 2 (“very true or often true”) and then used our previously-developed two-category qualitative coding scheme by classifying the teacher descriptions as either gender-related or non-gender-related. Examples of gender-related themes for Item 9 were “Obsessed with female actions, colors, activities,” “preoccupied with dressing up at house center,” and “Spiderman.” Examples of non-gender-related themes were “frequently day dreams,” “…food,” and “revengeful thoughts.” Corresponding gender-related theme examples for Item 66 were “Dresses up like a female” and “Drawing females” and non-gender-related themes were “paces” and “repeated cracking knees and elbows.” Two authors (ANN, JM) independently coded both items as either gender-related or non-gender-related. For Item 9 (n = 129), the kappa was .87 (*p* < .001); for Item 66 (n = 47), the kappa was .95 (*p* < .001). Unfortunately, it was not possible to code for qualitative comments in the referred and non-referred standardization samples because they were not available in the raw data file provided to us by Achenbach.

The present study constituted a reanalysis of data from previous research projects for which there was ethics approval from the [Centre for Addiction and Mental Health] Research Ethics Board. This research was conducted in accordance with the Declaration of Helsinki.

## Results

### Preliminary analyses

We first compared the gender-referred children for whom a TRF was completed vs. those for whom it was not (including the cases in which the TRF version for preschoolers was used). As expected, children for whom the TRF was completed were, on average, significantly older than those children for whom it was not, *t*(529) = 7.02, *p* < .001. There was no significant difference for year of assessment. Children for whom a TRF was completed had a significantly lower Full-Scale IQ (M, 101.1 vs. 108.4), came from a somewhat lower social class background (M, 42.1 vs. 46.8; absolute range 8–66) [42], and had higher Internalizing (M, 62.1 vs. 56.8) and Externalizing (M, 61.5 vs. 54.4) *T* scores on the CBCL (all *p* < .001). With age co-varied, these differences remained statistically significant, with the exception of social class.^5^

### Teacher ratings for Items 9 and 66

Table 1 shows the dichotomized teacher ratings for Items 9 and 66 (in percent) for the gender-referred children, the referred children, and the non-referred children, stratified by sex. For both the boys and the girls, the overall chi square test was statistically significant for both Items 9 and 66: Item 9 for boys, χ^2^(2) = 90.61, *p* < .00001; for girls, χ^2^(2) = 42.86, *p* < .00001; Item 66 for boys, χ^2^(2) = 42.21, *p* < .00001; for girls, χ^2^(2) = 16.28, *p* = .00029. To decompose the overall effect, three paired contrasts were conducted for both items: gender-referred vs. referred children from the standardization sample, gender-referred vs. non-referred children from the standardization sample, and referred vs. non-referred children from the standardization sample, by sex (Table 1).

**Table 1:** Teacher ratings of TRF Items 9 and 66 as a function of group and sex

Ratings of obsessions (Item 9)							
Groups	0		1 or 2		χ^2^(1)	*p*	OR (95% CI)
	n	%	n	%			
Boys							
Gender-referred vs.	172	58.3	123	41.7			
Referred	332	66.7	166	33.3	5.23	.022	1.43 (1.06–1.92)
Non-referred	433	86.9	65	13.1	82.45	< .001	4.76 (3.36–6.75)
Referred vs. non-referred					56.36	< .001	3.33 (2.41–4.58)
Girls							
Gender-referred vs.	49	62.0	30	38.0			
Referred	356	76.2	111	23.8	6.39	.011	1.96 (1.18–3.24)
Non-referred	414	88.7	53	11.3	35.12	< .001	4.78 (2.78–8.18)
Referred vs. non-referred					24.03	< .001	2.43 (1.70–3.47)

Ratings of compulsions (Item 66)							
Groups	0		1 or 2		χ^2^(1)	*p*	OR (95% CI)
	n	%	n	%			
Boys							
Gender-referred vs.	247	81.2	55	18.2			
Referred	415	83.3	83	16.7	< 1	ns	1.11 (.76–1.62)
Non-referred	473	95.0	25	5.0	34.90	< .001	4.21 (2.56–6.92)
Referred vs. non-referred					33.74	< .001	3.78 (2.37–6.03)
Girls							
Gender-referred vs.	72	90.0	8	10.0			
Referred	421	90.1	46	9.9	< 1	ns	1.01 (.46–2.24)
Non-referred	451	96.6	16	3.4	5.56	.018	4.90 (1.99–12.07)
Referred vs. non-referred					14.52	< .001	3.07 (1.71–5.52)

Referred and non-referred raw data from Achenbach and Rescorla [39] provided by Achenbach in an SPSS file

For Item 9, for the boys, it can be seen that teachers were significantly more likely to endorse this item with a rating of either a 1 or a 2 for both the gender-referred and referred samples when compared to the non-referred sample. It can also be seen that teachers were significantly more likely to endorse this item for the gender-referred boys than for the referred boys. For the girls, the findings were similar.

For Item 66, for the boys, it can be seen that teachers were significantly more likely to endorse this item with a rating of either a 1 or a 2 for both the gender-referred and referred samples when compared to the non-referred sample, but the comparison between the gender-referred boys and the referred boys in the standardization sample was not significant. For the girls, the findings were similar.

### Correlational analyses

In the gender-referred sample (collapsed across sex), we calculated the correlation between the continuous ratings for Items 9 and 66 for the TRF and the CBCL [36]. For Item 9, the mother-teacher correlation was .28 (n = 337, *p* < .001) and the father-teacher correlation was .23 (n = 248, *p* < .001). For Item 66, the mother-teacher correlation was .17 (n = 345, *p* = .002) and the father–teacher correlation was .11 (n = 255, *p* = .091). We also calculated the correlation between the continuous ratings for Items 9 and 66 and age (collapsed across sex), which were.11 (*p* = .029) and .00 (ns), respectively. For the referred sample, the correlations were .05 (ns) and −.07 (*p* = .033), respectively. For the non-referred sample, the correlations were −.01 and .02, respectively (both ns).^6^ Thus, age effects were either non-existent or extremely small.

### Qualitative analysis

For the qualitative analyses, teachers provided written comments for 84.3% (n = 129/153) of the gender-referred sample for whom Item 9 was rated as a 1 or a 2 and for 74.6% (n = 47/63) of the sample for who Item 66 was rated as a 1 or a 2 (see Table 1). For Item 9, 47.2% of the comments for boy were coded as gender-related compared to 30.4% for girls, a non-significant difference, χ^2^(1) = 1.52. For Item 66, the corresponding percentages were 32.4 and 0%, respectively, which was also not significant, χ^2^(1) < 1.

## Discussion

An emerging clinical and research literature has suggested a co-occurrence between GD and ASD (or ASD traits). VanderLaan et al. [36] had hypothesized that this link might be due, at least in part, to an elevated presence of intense/obsessional interests that involve gender-related behaviors. In their study, parents of gender-referred children endorsed CBCL Item 9 more frequently than they did for siblings and by parents in both referred and non-referred children from the CBCL standardization sample. This finding was, therefore, consistent with the proposition that the basis of the GD-ASD link is the tendency of gender-referred children to present clinically in a manner that corresponds to the ASD criterion pertaining to highly restricted and fixated interests. In this regard, it is important to note that this item corresponds very closely to two items on the SRS-2 that load on the RIRB subscale (Items 26: “Thinks or talks about the same thing over and over” and Item 31: “Can’t get his or her mind off something once he or she starts thinking about it”). The results for Item 66 also suggested that the ASD diagnostic criterion pertaining to repetitive behaviors and routines might also be relevant to GD in children. For this item, parental ratings were also elevated compared to siblings and non-referred children, but not when compared to referred children, so there was less support for a specificity effect. In relation to the SRS-2, this item bears some similarity to RIRB subscale Item 4: “When under stress…shows rigid or inflexible patterns of behavior…” In a comparative perspective, however, it could be argued that intense/obsessional interests (Item 9) provide a stronger basis than repetitive behaviors/routines (Item 66) for the link between GD and ASD.

Using the TRF, the present study provided a cross-validation of the CBCL findings [36]. For Item 9, the gender-referred children had significantly higher ratings than both the referred and non-referred children in the standardization sample but, for Item 66, the ratings were significantly higher only when compared to the non-referred children. Although the percentage of gender-referred children for which Items 9 and 66 were endorsed by teachers was lower than the percentage for which the items were endorsed by parents in VanderLaan et al. [36], the same was true for the referred and non-referred children. Also as in VanderLaan et al., gender-related themes were identified on both Items 9 and 66 for boys and, on Item 9, for girls as well. For example, on Item 9 for boys, 47% of the descriptors pertained to gender-related themes, which was similar to the percentage of 54% that mothers provided. Thus, the pattern across the two informants (parents, teachers) was very similar.

If there is, indeed, an empirical basis for the role of gender-related obsessionality that contributes to the GD-ASD link, the possible developmental pathways need to be formulated. As noted earlier, one idea is that ASD sometimes leads to intense interests in cross-sex objects or activities, giving rise to a clinical presentation of GD. Thus, on this basis, one would predict that GD children would also exhibit additional features of ASD. In the study by Skagerberg et al. [23], this appeared to be the case: although Skagerberg et al. did not provide formal statistical tests, our own analysis of their data showed that, compared to a normative sample, children and adolescents with GD had significantly higher ratings on all of the other subscales of the SRS, not just the one pertaining to restricted interests and repetitive behaviors.^7^

The Skagerberg et al. [23] data would appear to challenge another developmental pathway proposed by VanderLaan et al. [36]. If restricted and intense cross-sex interests are simply a manifestation of GD, the ASD “flavor” might be only subclinical or even superficial, because the intensity of the interests is only a marker of the GD and not an underlying ASD. If such were the case, then few, if any, additional ASD features should accompany intense cross-sex interests. But this was clearly not the case in the Skagerberg et al. data set.

From Skagerberg et al. [23] and other systematic studies of GD samples (noted earlier), it is clear that there are many children with GD who would not be diagnosed with an ASD or would even be in the clinical range on dimensional measures of ASD traits, as, for example, on the SRS. Recognition of this variability is consistent with the principle of equifinality [43]. ASD or ASD traits, including the presence of intense and restricted interests, may lead to gender dysphoria, but for those GD children without ASD or ASD traits the presence of intense and restricted interests may be caused by other underlying processes. This would, of course, be consistent with multifactorial models of gender dysphoria, in which the relative contribution of risk factors will vary in their relative weight from one child to the next [44]. Along similar lines, it should also be noted that there are now several studies which document an elevation in ASD traits, as measured by the SRS, in children referred for a variety of clinical problems [45–49], not just in children referred for GD, which clearly points to a pattern of non-specificity.

This non-specificity effect is a clear indication that the hypothesized GD-ASD link requires a more nuanced examination. One such strategy would be to design formal tests of equifinality in which GD children are divided into two subgroups: those with ASD or ASD traits and those without. One could then examine whether or not the two subgroups differ in other important ways. In one study, VanderLaan et al. [50] reported in a sample of children with GD that those with higher ASD traits and a higher score on a dimensional measure of gender-variant behavior had a higher birth weight. VanderLaan et al. [50] noted that high birth weight has been identified as a risk factor for ASD and that it is also associated with lower prenatal levels of testosterone in males and with masculinized somatic features, such as a greater anogenital distance, in females. This finding is consistent with one study that reported an association between the degree of demasculinizing endocrine disruptor chemicals in maternal blood and ASD traits in children [51]. In another study, Shumer et al. [52] found that mothers (but not fathers) in the Nurses’ Health Study II and the Growing Up Today Study 1 who had higher self-reported SRS scores rated their children as higher in gender-variant behaviors, suggesting some type of underlying biological liability, perhaps along the maternal line, for both variables. These two studies lend some support for further tests of the equifinality principle with regard to the GD-ASD link.

## Limitations

There are four limitations to the current study that should be noted. First, we assessed the focal variables of obsessional interests and repetitive behaviors using only single items from the TRF and our primary analysis was based on a dichotomous (present vs. absent) metric. Although both our prior CBCL analysis and the current TRF analysis were quite successful in detecting significant between-groups effects, we recognize that dimensional measures, such as the SRS, would be psychometrically superior as this line of research continues. However, given the current intense interest in the GD-ASD link in the literature, it was our view that the use of a large “archival” data set (i.e., using a sample of children going back several decades) would add to this contemporary discourse. Second, although we were able to obtain TRFs on 73% of the entire sample of gender-referred children assessed between 1986 and 2013, we were not able to use the TRF data that were available for preschoolers because the relevant items are not on this version. Thus, future research should use the SRS so that the restricted interests and repetitive behaviors construct can be evaluated during the developmental period in which GD is often first expressed [1]. Third, it should be considered whether or not parents and teachers who endorsed Items 9 and 66 and provide gender-related themes were “over-reacting” because the child’s gendered behavior was atypical or if the ratings represent bona fide evidence of obsessionality and compulsivity. On this point, one could test this by looking at children whose parents describe them as being preoccupied with gender-typical behaviors, as in the Halim et al. [6] study of the “pink frilly dresses” phenomenon in young girls and to see if they too would be more likely to endorse these items when compared to girls who are not seen as overly preoccupied with gender normative behaviors. Lastly, it should be emphasized that our data speak more to the potential presence of ASD traits than to the categorical ASD diagnosis.

We recognize that our data only speak to one aspect of an ASD but not other core elementss, such as marked impairment in social communication and social interaction. Thus, we in no way wish to argue that elevations in obsessional interests/behaviors per se are sufficient in making any kind of definitive conclusion about ASD. However, it is important to note that our data are consistent with one study that analyzed CBCL and TRF items that discriminated children with an ASD diagnosis from clinic-referred children classified as having an internalizing disorder, an externalizing disorder, no diagnosis, and children from the general population [53]. So et al. [53] found that 10 CBCL/TRF items were significantly higher in the ASD group than the other four groups: Items 9 and 66 were two of these items, with between-groups odds ratios ranging from 1.25 to 2.08 for the 10 CBCL items and 1.17–1.55 for the 10 TRF items. Given these findings, it is our view that Items 9 and 66, at least in children, may be more suggestive of ASD traits than traits suggestive of an Obsessive–Compulsive Disorder because natural history data suggest that OCD onsets at a much later age than ASD [1].

To date, the GD-ASD literature in children has been largely limited to case reports. Other than our own work [36, 49], only the Skagerberg et al. [23] study used a dimensional assessment measure to assess putative ASD traits and only one study, which used a selective sub-sample of children and adolescents referred for gender dysphoria, employed a structured diagnostic interview schedule to ascertain an autism diagnosis [20]. Going forward, researchers in this specialty area will need to decide if there would be benefits in using more formal diagnostic methods, such as the Autism Diagnostic Observation Schedule [54], to ascertain the percentage of children referred for gender dysphoria who would meet criteria for the diagnosis.

## Conclusion

Our TRF study provides a cross-validation of our previous CBCL study of an elevation in intense interests/obsessional traits among children referred for gender dysphoria as compared to both referred and non-referred children in the standardization sample and, to a lesser extent, with regard to repetitive behaviors. These findings, therefore, give some support to the idea that there may be a link between gender dysphoria and ASD traits. However, the emerging literature that suggests a non-specific pattern of elevations in ASD traits among clinic-referred children in general calls for a more focused examination of why such a link may be present among at least some children with a DSM diagnosis of gender dysphoria.

## Abbreviations

ASD: Autism Spectrum Disorder
CBCL: Child Behavior Checklist
DSM: Diagnostic and Statistical Manual of Mental Disorders
GD: gender dysphoria
GID: Gender Identity Disorder
SRS: Social Responsiveness Scale
TRF: Teacher’s Report Form

## Footnotes

1 We will use primarily GD to reflect the current DSM-5 diagnostic label, but use GID when it is historically accurate to do so (e.g., regarding the clinical diagnosis of the participants in this study).

2 In developmental clinical psychology and psychiatry, the CBCL [37] is one of the most widely used parent-report measures of behavioral and emotional problems in children and adolescents. It contains a total of 118 items, each of which is rated on a 0–2 point scale for frequency of occurrence. Factor analysis has identified both broad-band (Internalizing, Externalizing) and eight narrow-band dimensions of behavioral and emotional disturbance (e.g., “Anxious/Depressed,” “Aggressive Behavior.” Items 9 and 66 load on the “Thought Problems” narrow-band scale, which is part of a suite of three narrow-band dimensions that do not load on either the Internalizing or Externalizing broad-band dimensions. On average, completion of the CBCL takes about 15–17 min [37, p. 14].

3 The TRF [38] is similar in design and format to that of the CBCL. There are 25 items on the TRF that are more appropriate for the school setting (e.g., “Dislikes school”) and these items replace 25 items on the CBCL. Factor analysis has identified the same broad-band and narrow-band dimensions of behavioral and emotional disturbance as on the CBCL. The behavioral and emotional problem items on the TRF can be completed, on average, in about 10 min [38, p. 11].

4 Our clinic began administering the TRF in 1986, when it was first published [40]. For preschoolers, the Caregiver-Teacher Report Form for Ages 1–1/2–5 was administered once it became available [41]; unfortunately, this version of the TRF does not contain the two items analyzed in this study.

5 These analyses are available from the corresponding author upon request.

6 It was not possible to calculate mother–teacher correlations for Items 9 and 66 in the standardization samples because the raw data for the CBCL and TRF were in separate SPSS files.

7 We conducted *t* tests on the data provided in Table 2 in Skagerberg et al. [23]. These analyses are available from the corresponding author upon request.
